# Supplementary material for: metaVaR: Introducing metavariant species models for reference-free metagenomic-based population genomics
Source: PLoS One. 2020 Dec 30;15(12):e0244637. doi: 10.1371/journal.pone.0244637 (PMC7773188; doi:10.1371/journal.pone.0244637)
Supplement: S1 File — (PDF) [file pone.0244637.s001.pdf]

S1 File
metaVaR: introducing metavariant species models for reference-free metagenomic-based population genomics

November 23, 2020

1 Supplementary Method 1

Data: $G(V, E, W)$
Result: $MWIS$
$MWIS = \emptyset$;
while $G \neq \emptyset$ **do**
  foreach cc **do**
    foreach $v_i \in cc$ **do**
      if $f(v_i) = argmax(f)$ **then**
        $MWIS = MWIS \oplus \{v_i\}$;
        $G = G \setminus \{v_i, neighbors(v_i)\}$;
        break;
      end
    end
  end
end

Algorithm 1: WMIN algorithm from Sakai et *al.*

2 Supplementary Results 1

Raw results of the application of metaVaR on real large metagenomic data set

Size_Fraction	Nmb_samples	Total_Number_Metavariants	After Filtering	Number_MVC	Number_MVS	Size_Range_MVS
0,8-5µm	25	6,920,311	970.553	959	33	151-1,767
5-20µm	27	3,173,822	141.475	686	18	175-1,578
20-180µm	31	6,207,130	1,136,400	1.045	26	114-1,886
180-2000µm	31	7,497,058	311.304	1.53	36	119-1,238
