# Supplementary material for: metaVaR: Introducing metavariant species models for reference-free metagenomic-based population genomics
Source: PLoS One. 2020 Dec 30;15(12):e0244637. doi: 10.1371/journal.pone.0244637 (PMC7773188; doi:10.1371/journal.pone.0244637)
Supplement: Attachment — Submitted filename: Rebuttal_letter.pdf [file pone.0244637.s002.pdf]

Rebuttal letter for "metaVAR: introducing metavariant species models for reference-free metagenomic-based population genomics"

We first acknowledge the reviewers for the interesting points they spotted and the questions they raised. In the following document, we will answer to reviewers point by point.

Reviewer 1

> In the article "metaVAR: introducing metavariant species models for reference-free metagenomic-based population genomics", the authors present a method to retrieve polymorphism data from metagenome samples without the need for a reference genome, based on performing variant calling directly on the raw reads, then grouping the different variants into "Metavariant Species" (MVS) based on the variant coverage.
>
> The method is mathematically sound and it certainly has a place in current metagenomic analyses. Although the production of Metagenome Assembled Genomes (MAGs) is widespread on the field, there are still a lot of samples for which the assembly of contigs to build MAGs is still difficult (One such example is any sample where one species of microorganism is specially abundant, such as salterns). However, I see a few issues that need to be addressed before this article can be published.

Reply: Thank you for your positive comments on the paper with regards to its usefulness to the community.

Reviewer Point P 1.1 — One of them is the overall readability of the article, specially the introduction. Although there are not any grammatical or spelling errors, there are some phrases that should be rewritten to make it easier to understand. I would bring special attention to lines 11-16, 50-53 and 256-275, but the entire article could do with an additional punctuation pass. The figures could do with a bit of retouching: Figure 1 in particular has too much information, including pictures referring to different sections of the article. Offloading some of the sub figures to Supplementary data would make the figure easier to parse. I am also missing a figure explaining in a graphical manner the concept of variable loci and metavariants: understanding these concepts is vital to understand the method, but the definition in the text is correct but a tad too formal for someone without proper mathematics formation, and a good figure could help biologists understand the core concepts of the article. The Algorithm 1 could also be removed from the article, as it is not required to understand the method and could be moved to Supplementary Data.

Reply: Thank you for noting this point. The sentences that were a bit too long were reformatted to allow a better and comfortable reading. According to your comments, we corrected the different features that would help making the manuscript clearer and lighter.

Reviewer Point P 1.2 — My main gripe with the article, however, is the application of this method to real metagenomic analyses. I'm particularly worried about the following issues: The scoring of each metavariant cluster depends on the number of samples, but metagenomic studies

do not usually include replicates and the amount of samples sequenced is usually very low. How does sample size affect metavariant clustering?

Reply: Thank you for challenging us on this particular point related to the evaluation of the method performances. To address them rigorously, we performed several metagenomes sampling (from 3 to 6 metagenomes) and run metaVaR on the selected samples, then we measured the metaVaR performances on each subsampling (i.e number of MVS, recall, precision, purity and entropy).

We showed that in our simulation setup, the precision and purity increase with the number of metagenomes while the recall and entropy decrease with number of samples. High precision and purity are reached with at least 6 metagenomes. The fact that the recall drops whith increasing number of metagenomes is due to SNV filtering. Indeed, the more samples are added to metaVaR, the more SNVs get filtered out because they do not reach the minimum coverage in at least one sample. Pushing this idea forward could lead to the very low recall for a very high number of metagenomes (>100) which could end up with no MVS (at least 100 SVNs should be present in a MVS to be valid). This shows that the number of metagenomic samples has an important role in the balance between precision and recall. The methods (L258), results (L306), discussion (L425) and figure (figure 5) concerning the analysis of the sample size effects on metavaR performances were added to the manuscript.

Reviewer Point P 1.3 — How many samples are needed to obtain a good separation of clusters?

Reply: This is a very good but tricky question. Unfortunately, we cannot give a general number of samples that will always ensures a good separation of the clusters without data dependency. However, using our metagenome sampling analysis, we showed that the six MVSs corresponding to the six initial species can be found only once when using 5 samples and most of the time when using more than 5 metagenomic samples. Also it is important to note that as seen in P1.2 a high number of samples will also lower the recall. This point is now discussed L425.

Reviewer Point P 1.4 — Does the homogeneity of the samples (How similar are they to one another, in terms of population composition) affect the scoring somehow?

Reply: The general answer to this question is yes, the homogeneity of the samples will greatly affect the quality of the MVS. This point is not dependent of our method but is a general issue in clustering. Two individuals having similar features will tend to be clustered together, inversely, a high disparity in the sample composition will greatly help separating the MVS. To avoid this clustering issue, we can advise to use a high number of samples. In our simulation setup, when sampling 4 metagenomes, Yersinia pestis (NC_003143.1) and Escherichia coli (NC_000913.3) have very similar pattern (see figure 3) and they differ mostly in metagenome7. This explained why their specific MVSs have a high precision only by using more than 5 samples including metagenomes7 that discriminates them well (See figure 3). Unfortunately, in real cases we don't know how homogenous two samples are. So we can only advise to increase the number of samples which will, again, decrease the recall, however we can consider that more than hundreds of SNPs are still very informative for population genomics. This point is know discussed L427.

Reviewer Point P 1.5 — I am particularly troubled about the number of metavariants the method is able to recover: the simulated metagenomic dataset test only uses 6 genomes, and the real metagenomic test focuses only on a single genome. A real metagenomic sample is going to have a lot more genomes. How many is the tool able to recover?

Reply: This question is legitimate, and it is true that it is difficult to provide a straightforward answer based on the present work. However, we were able to run metaVaR on four real large datasets of metagenomic variants (more than 18 millions of variants in total), that already produced in a previous work using *DiscoSNP++* (see Discovering Millions of Plankton Genomic Markers from the Atlantic Ocean and the Mediterranean Sea Arif et al. 2018), and were able to retrieve a total of 113 MVSs (the analysis of these 113 MVS is the subject to another study). We discussed this aspect L446 and provide supplementary results (Supplementary Table1) that can give a glimpse of metaVaR applications on real large metagenomic data.

Reviewer Point P 1.6 — If the tool is only able to recover a limited number of genomes per run, is it possible to direct the tool to recover an specific genome?

Reply: Indeed, *DiscoSNP++* has the ability to call variants from metagenomic variants using a reference genome. If one is interested to find a particular species in a set of metagenomes, *DiscoSNP++* can be used in this way to retrieve variants belonging to this targeted species. For this particular task, we refer directly to our previous study which demonstrated how *DiscoSNP++* can be used for population genomic analysis from metagenomic data with a specific targeted genome/transcriptome (Arif, M, Gauthier, J, Sugier, K, et al. Discovering millions of plankton genomic markers from the Atlantic Ocean and the Mediterranean Sea. Mol Ecol Resour. 2019; 19: 526– 535. https://doi.org/10.1111/1755-0998.12985) We also drive the reader toward this study in the manuscript.

Reviewer Point P 1.7 — Although I understand the motivation of building MVS, a biologist using this tool still needs a way to connect a MVS to a genome, which the article does not include.

Reply: Raising this point is very interesting, thank you for giving a chance to clarify how MVS can be connected to genomic resources.

It could have been unclear in the text, but when using *DiscoSNP++*, it generates the sequences carrying the variants (clarified in line 81). Thus, if the user thinks his favorite species is present in the samples and wants to identify the cluster corresponding to it, the user can map the metagenomic reads corresponding to the variants on the species' genome/transcriptome and see if the MVS sequences align on it. An example of this process is presented in the test on the real data, where we were able to recover metavariants that specifically mapped on the genome of the zooplankton species *Oithona nana* using the classical alignment method bwa-mem.

To date, our team already tested the dbscan clustering method in a previous work (see Investigating population-scale allelic differential expression in wild populations of *Oithona similis* (Cyclopoida, Claus, 1866; Laso-Jadart et al . Ecol Evol 2020). In this study, the goal was to extract the variants belonging to *Oithona similis*, a species very abundant in Arctic Seas. After using dbscan with a specific couple of parameters on seven Arctic *Tara* Oceans samples, we were able to obtain one cluster containing around 25,000 variants that successfully mapped on newly assembled transcriptomes for 97% of them. These results pushed us to improve the method and publish it on its own to be available for the community.

Indeed, in complex communities comprising eukaryotes such as small algae or small animals, as reference genomes are rare, the method would greatly help by avoiding the laborious task of generating genomes or transcriptomes. In the absence of a genome, it would also be possible to link the sequences with public databases (MMETSP, ncbi) and retrieve specific taxonomic assignations. Biologists can work with taxonomical assignation of the MVS with various level of accuracy as for OTU in metabarcoding.

At the end, mapping metavariants on genomes is an open option that can be performed on genomic/transcriptomic user database using bwa/bowtie. To clarify this point, we explain how MVS can be linked to genome or transcriptome sequences at L396-402.

Reviewer Point P 1.8 — If these issues are adressed, then I have no doubt metaVaR has a bright future as a tool in population genomics.

Reply: Thank you very much for this comment and for all your questions that really helped us to improve the quality of our manuscript. We firmly hope as well that this method will be helpful for researchers that use metagenomic data and have a great interest in population genomics.

Reviewer 2

> In this manuscript the authors introduce a new method to detecting micro-diversity in metagenomic datasets. They also introduce the concept of metavariants and metavariant species. Their tool, MetaVaR was tested on both real and simulated datasets and presented superior performance. The manuscript is relevant and well written, the experiments are well designed and the results support the conclusions. Nevertheless some minor changes and clarifications are necessary to make this manuscript suitable for publication.

Reply: We thank you for your comments, we particularly appreciate that you pointed the writing of the article and the design of the study.

Reviewer Point P 2.1 — Ln 71: It seems to me that considering only a single metavariant might be a major limitation of the proposed method, albeit necessary to make the computations feasible. This should be mentioned in the discussion.

Reply: This is an interesting point. We agree that it is true that only one metavariant by sequence is a rather restricting choice, but helps for the computation based on the allele frequency instead of multi-allele frequencies. This is now mentioned in discussion, line 386.

Reviewer Point P 2.2 — Ln 73: Would it not be more logical to establish the reference as the variant that has higher coverage?

Reply: In a biological point of view yes, we completely agree. However, this setting was fixed by *DiscoSNP++* where the lexical order was an arbitrary choice. However, the user can easily change the reference/alternative variants afterwards based on the coverage of each of the alleles directly in the VCF file.

Reviewer Point P 2.3 — Ln 113: Which distance metric was used with the DBSCAN algorithm? Did you test different metrics?

Reply: The default and inherent distance used in DBSCAN is the euclidean distance, and no other distances were tested. This is now added in the manuscript for accuracy, lines 113.

Reviewer Point P 2.4 — Ln 189-191: This setup is an overly simplistic representation of metagenomes. In most real world datasets metagenomes are made up of many more species and often multiple strains of the same species. Using only six species and no multiple strains from the same species is likely to inflate the precision and skew the other evaluation metrics.

Reply: Yes, we agree that the simulated dataset is a simple representation of the reality. Nonetheless in our mock, each species contains two strains, that derived from 97% or 98% identity from each other and are present in various proportions. The objective is here to show how the method works with simple metagenomes, and to compare it with existing clustering methods.

Reviewer Point P 2.5 — Ln 195: Perhaps the term "communities" is more adequate than "populations" considering you are dealing with different species.

Reply: Indeed, this has now been changed, line 197.

Reviewer Point P 2.6 — Figure 1C: Is there evidence to support that the DBSCAN noise points are all the result of inter-species variation or is this one of your assumptions? In case of the latter, this should be explicitly stated along with the rationale behind it.

Reply: Indeed, noise points can be inter-species variants, sequencing errors or variants with specific pattern of coverage, unrelated to the genome it belongs to. We thus modified it for some clarity and to avoid misleading statement, from "inter-species variants" to "unclustered variants" in the legend of figure 2B.

Reviewer Point P 2.7 — Figure 2A: Is it correct to assume that coverage values refer to specific positions within genomes rather than coverage of the whole genome? If so, please clarify

Reply: Exactly, it is now stated as "variable loci genomic coverage" in Figure 3A.

Reviewer Point P 2.8 — Ln 253: word missing after evolutionary?

Reply: Indeed, modified from "evolutionary" to "pattern", line 255
